# Effects of ventilatory strategy on arterial oxygenation and respiratory mechanics in overweight and obese patients undergoing posterior spine surgery

**DOI:** 10.1038/s41598-019-53194-2

**Published:** 2019-11-12

**Authors:** Kyung Mi Kim, Jung Ju Choi, Dongchul Lee, Wol Seon Jung, Su Bin Kim, Hyun Jeong Kwak

**Affiliations:** 1 Department of Anesthesiology and Pain Medicine, Asan Medical Center, University of Ulsan College of Medicine, Seoul, Republic of Korea; 2 Department of Anesthesiology and Pain Medicine, Gil Medical Center, Gachon University College of Medicine, Incheon, Republic of Korea

**Keywords:** Outcomes research, Risk factors

## Abstract

Prolonged inspiratory to expiratory (I:E) ratio ventilation may improve arterial oxygenation or gas exchange and respiratory mechanics in patients with obesity. We performed a randomised study to compare the effects of the conventional ratio ventilation (CRV) of 1:2 and the equal ratio ventilation (ERV) of 1:1 on arterial oxygenation and respiratory mechanics during spine surgery in overweight and obese patients. Fifty adult patients with a body mass index of ≥25 kg/m^2^ were randomly allocated to receive an I:E ratio either l:2 (CRV; n = 25) or 1:1 (ERV; n = 25). Arterial oxygenation and respiratory mechanics were recorded in the supine position, and at 30 minutes and 90 minutes after placement in the prone position. The changes in partial arterial oxygen pressure (PaO_2_) over time did not differ between the groups. The changes in partial arterial carbon dioxide pressure over time were significantly different between the two groups (P = 0.040). The changes in mean airway pressure (Pmean) over time were significantly different between the two groups (P = 0.044). Although ERV provided a significantly higher Pmean than CRV during surgery, the changes in PaO_2_ did not differ between the two groups.

## Introduction

The overweight and obese population has increased sharply in recent years^1^. This is both a concern for general healthcare and a major risk factor for related diseases and perioperative morbidity^2^. Several studies have revealed that obesity is related to poor postoperative outcomes, such as longer hospital stay, high incidence rates of wound infection, and pulmonary complication^3–5^.

Obesity itself leads to abnormalities of lung mechanics and function. Thus, the obese population has a high incidence of postoperative pulmonary complications resulting from reduced lung compliance, decreased functional residual capacity, and susceptibility to atelectasis formation under general anaesthesia^4^. Cautious management of mechanical ventilation is necessary in patients with obesity to control these altered respiratory mechanics, which lead to impaired oxygenation and gas exchange during surgery.

Moreover, obesity correlates with an increased risk of intraoperative hypoxaemia^6^. Numerous studies have focused on methods to improve oxygenation or respiratory mechanics in patients with obesity during the perioperative period^5,7–10^, and lung protective strategies have been employed in such patients undergoing general anaesthesia^11^. Conventionally, high positive end expiratory pressure (PEEP) with the recruit manoeuvre is used to manage impaired oxygenation in patients with obesity during general anaesthesia^8^. Prolonged inspiratory to expiratory (I:E) ratio ventilation, which is also an effective ventilatory manoeuvre in obese patients undergoing laparoscopic bariatric surgery^12,13^; this technique improves gas exchange, arterial oxygenation, and respiratory mechanics in patients with acute respiratory distress syndrome or acute lung injury. A prolonged I:E ratio sustains increased alveolar pressure and decreases dead space, consequently improving arterial oxygenation and respiratory function.

Therefore, we hypothesized that prolonged I:E ratio ventilation improves arterial oxygenation and respiratory mechanics in overweight and obese patients undergoing lumbar spine surgery. This randomised study compared the conventional ratio ventilation (CRV) of 1:2 with the equal ratio ventilation (ERV) of 1:1 in terms of their effect on arterial oxygenation and respiratory mechanics during posterior lumbar spine surgery in overweight and obese patients placed in the prone position.

## Results

### Study population

**Figure 1:**
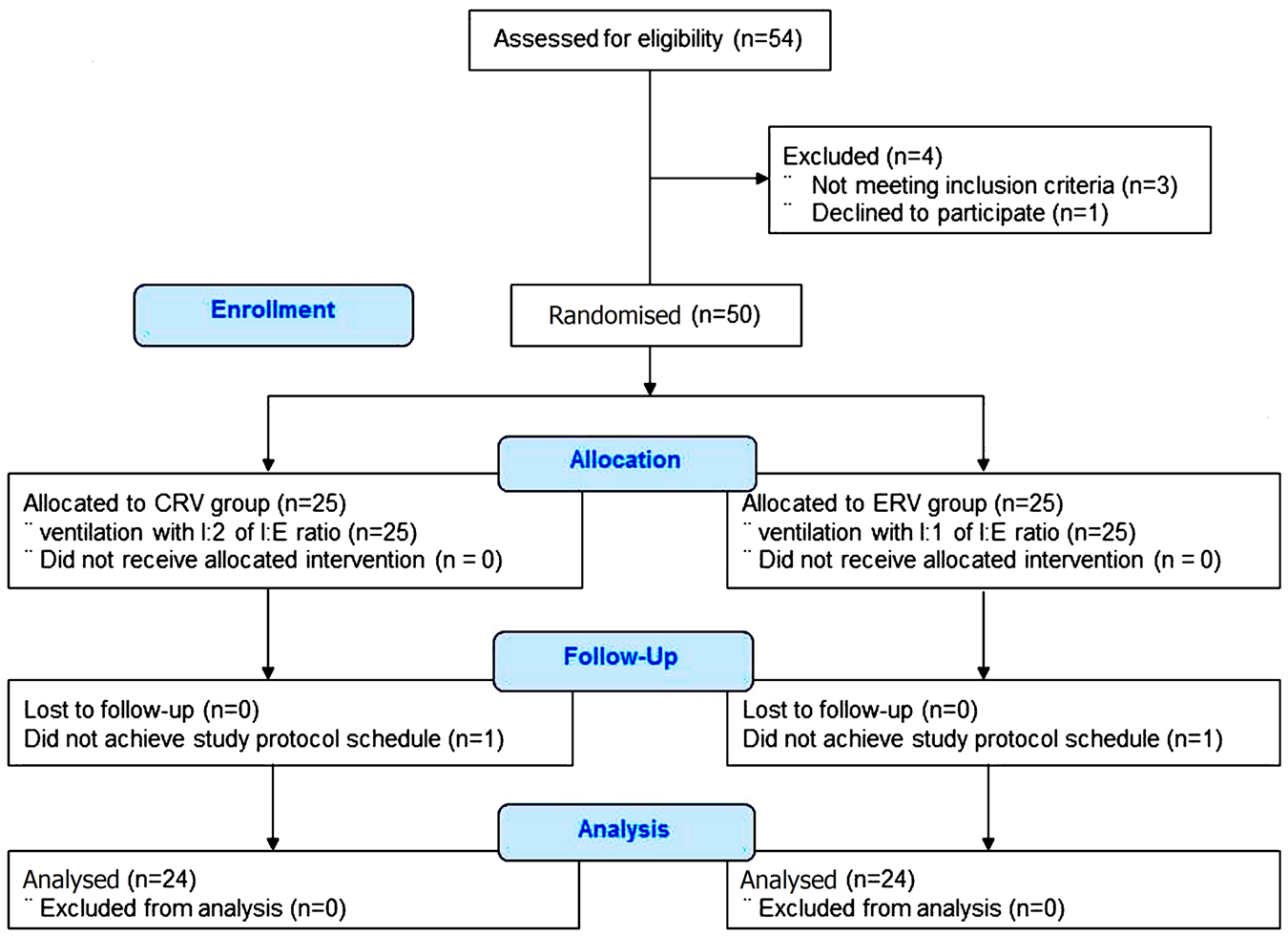
Enrolment of patients undergoing lumbar spine surgery. Between November 2016 and October 2018, 54 patients who underwent lumbar spine surgery in our institution were assessed. Four patients did not meet the inclusion criteria and two patients dropped out. Thus, 48 patients were subjected to the study protocol.

Fifty patients were enrolled in the present study; two dropped out, one in each group, because they were not subjected to the scheduled study protocol (Fig. 1). Demographic variables are shown in Table 1.

**Table 1:** Demographics.

	CRV (n = 24)	ERV (n = 24)	P-value
Age (year)	52.3 ± 10.0	54.6 ± 9.8	0.420
Gender (male)	18 (75%)	13 (54.2%)	0.227
BMI (kg/m^2^)	28.7 ± 2.7	27.4 ± 1.9	0.055
Operation time (min)	185.2 ± 88.4	180.4 ± 100.8	0.862
Prone time (min)	198.3 ± 84.9	199.6 ± 100.3	0.963
Anaesthesia time (min)	229.0 ± 90.3	235.6 ± 101.5	0.811
Admitted fluid (mL)	1277.0 ± 622.8	1412.5 ± 1188.6	0.623

Values are means ± SD or number of patients (%).

CRV = Patients administered 1:2 ratio ventilation; ERV = Patients administered equal ratio ventilation; BMI = body mass index.

### Analyses of arterial blood gas

**Figure 2:**
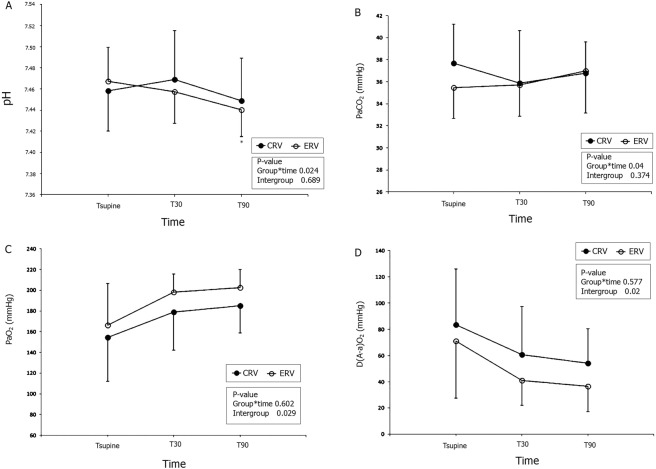
Changes in arterial blood gas values during surgery. The changes in pH (**A**) and PaCO_2_ (**B**) over time were significantly different between CRV (•, filled circle) and ERV (○, empty circle) groups. PaO_2_ (**C**) was significantly higher, whereas D(A-a)O_2_ (**D**, alveolar to arterial oxygen tension gradient) was significantly lower in the ERV group than in the CRV group. In the ERV group, PaO_2_ was significantly higher at T30 and T90 than at Tsupine, whereas there was no difference in the CRV group. CRV = Patients administered 1:2 ratio ventilation; ERV = Patients administered equal ratio ventilation; Tsupine = After induction of anaesthesia in the supine position; T30 and T90 = At 30 and 90 min after placement in the prone positioning, respectively; pH = pH of arterial blood gas analysis; PaO_2_ = Partial arterial oxygen pressure; PaCO_2_ = Partial arterial carbon dioxide pressure. ^*^P < 0.05 vs. Tsupine within the group.

Figure 2 illustrates changes in arterial blood gas during surgery. The changes in arterial pH over time were significantly different between the two groups (P = 0.024). In the ERV group, arterial pH was significantly lower during surgery (P < 0.001), and it was lower at 90 minutes (T90) after placement in the prone position than in the supine position (Tsupine) (P < 0.001). The changes in partial arterial carbon dioxide pressure (PaCO_2_) over time were significantly different between the two groups (P = 0.040). However, PaCO_2_ was not significantly changed during surgery in either group. The two groups did not differ in terms of the changes in oxygen partial pressure (PaO_2_) or alveolar-to-arterial oxygen gradient (D(A-a)O_2_) over time, although PaO_2_ was significantly increased and D(A-a)O_2_ significantly decreased during surgery in both groups (P < 0.001 in both cases).

### Respiratory mechanics

Table 2 lists the patients’ respiratory mechanics during surgery. The changes in peak airway pressure (Ppeak), driving airway pressure (Pdriving), dynamic lung compliance (Cdyn), and static lung compliance (Cstatic) over time were not different between the two groups. While Ppeak was not significantly changed, Pdriving was significantly increased during surgery in both groups (P = 0.008 in the CRV group and P < 0.001 in the ERV group). In both groups, there were significant decreases in Cdyn (P < 0.001 in both cases) and Cstatic (P = 0.025 in the CRV group and P < 0.001 in the ERV group). The changes in mean airway pressure (Pmean) over time were significantly different between the two groups (P = 0.044). Pmean was significantly lower at 30 minutes after in the prone position (T30) and T90 than at Tsupine in the CRV group only (P = 0.001 in both cases).

**Table 2:** Intraoperative respiratory mechanics.

		Tsupine	T30	T90	P-value	
					Group*time	Intergroup
Ppeak (cmH_2_O)	CRV	21.4 ± 3.6	20.9 ± 2.7	21.1 ± 2.6	0.535	<0.001
	ERV	17.9 ± 1.8	18.1 ± 2.0	18.1 ± 1.9		
Pdriving (cmH_2_O)	CRV	12.7 ± 2.9^*^	13.9 ± 1.7	14.6 ± 2.2^*^	0.600	<0.001
	ERV	10.4 ± 1.6^*^	12.3 ± 1.8^*^	12.8 ± 1.8^*^		
Pmean (cmH_2_O)	CRV	8.3 ± 1.3	7.5 ± 1.1^*^	7.4 ± 1.0^*^	0.044	0.015
	ERV	8.5 ± 1.2	8.4 ± 1.3	8.5 ± 1.1		
Cdyn (mL/cmH_2_O)	CRV	47.8 ± 13.0	31.6 ± 5.9^*^	31.4 ± 6.6^*^	0.347	0.018
	ERV	56.9 ± 15.6	35.5 ± 6.5^*^	35.5 ± 6.5^*^		
Cstatic (mL/cmH_2_O)	CRV	47.8 ± 13.0	42.2 ± 8.3	40.9 ± 8.3	0.093	0.088
	ERV	56.9 ± 15.6	45.0 ± 7.7	43.8 ± 8.9		

Values are means ± SD.

Ppeak = Peak inspiratory pressure measured by anaesthesia machine; Pdriving = Driving airway pressure (plateau pressure − PEEP); Pmean = Mean airway pressure measured by anaesthesia machine; Cdyn = Dynamic lung compliance: tidal volume/(Ppeak - PEEP); Cstatic = Static lung compliance: tidal volume/Pdriving; CRV = Patients administered 1:2 ratio ventilation; ERV = Patients administered equal ratio ventilation; Tsupine = After induction of anaesthesia in the supine position; T30 and T90 = At 30 and 90 min after placement in the prone positioning, respectively.

^*^P < 0.05 vs. Tsupine within the group.

### Haemodynamic signs

The haemodynamic parameters during surgery are listed in Table 3. The changes in mean arterial pressure and heart rate over time were not different between the two groups.

**Table 3:** Haemodynamic parameters.

		Tsupine	T30	T90	P-value	
					Group*time	Intergroup
MAP (mmHg)	CRV	89.5 ± 12.7	80.7 ± 9.8^*^	80.9 ± 11.7^*^	0.336	0.616
	ERV	87.5 ± 15.7	83.4 ± 13.8	84.5 ± 11.6		
HR (bpm)	CRV	78.7 ± 14.4	67.8 ± 13.7^*^	67.6 ± 12.5^*^	0.978	0.976
	ERV	78.2 ± 14.5	67.8 ± 10.8^*^	67.8 ± 10.1^*^		

Values are means ± SD.

MAP = Mean arterial pressure; HR = Heart rate; CRV = Patients with 1:2 ratio ventilation; ERV = Patients with equal ratio ventilation; Tsupine = After induction of anaesthesia in the supine position; T30, T60, and T90 = At 30, 60, and 90 min after placement in the prone position, respectively.

^*^P < 0.05 vs. Tsupine within the group.

## Discussion

The current study, which was expanded from our previous study assessing the effect of prolonged inspiratory ventilation in patients undergoing laparoscopic bariatric surgery^13^, suggests that ERV does not confer superior arterial oxygenation than CRV during posterior lumbar spine surgery in overweight and obese patients placed in the prone position under general anaesthesia. However, ERV did confer a significantly higher Pmean than CRV during surgery. This was the first study to reveal the impact of prolonged inspiratory ventilation on patients in the prone position.

Postoperative pulmonary complication is one of the main factors determining length of hospital stay and cost burden on patients. Generally, general anaesthesia using muscle paralysis generates impaired pulmonary gas exchange because of alterations in respiratory mechanics and atelectasis formation. Obesity may increase the risk of postoperative pulmonary complications^14^. Consistent with a previous study^13,15^, our results showed that PaO_2_ was significantly higher at T30 and T90 than at Tsupine in the ERV group, and that changes in Pmean were higher in the ERV group than in the CRV group, but this latter effect did not reach statistical significance. ERV improves oxygenation by increasing Pmean and concomitantly reducing Ppeak^13^. Pmean is related to arterial oxygenation because it is the average alveolar pressure that opens and inflates the alveoli against the elastic recoil of the lungs and chest wall^16^. An elevated Pmean value induces alveolar recruitment, which improves ventilation perfusion mismatch and blood oxygenation^17^. Thus, a prolonged I:E ratio sustains an increased alveolar pressure, thus increasing patients’ functional residual capacity^18^. Although our results do not confirm that ERV is clinically superior to CRV, ERV may be feasible in overweight and obese patients with deteriorated oxygenation during general anaesthesia.

PaO_2_ significantly increased over time in both the ERV and CRV groups, perhaps because the patients were placed in the prone position. Numerous studies have demonstrated that the prone position can improve oxygenation in pneumonia, acute respiratory distress syndrome, and acute lung injury^19–21^. Pelosi *et al*. also reported that the prone position resulted in improved lung compliance, a rise of functional residual capacity, and improved oxygenation in obese patients under elective general anaesthesia^22^. A rise in functional residual capacity was also seen after placement of the patients in the prone position, perhaps because there was decreased cephalad pressure on the diaphragm and changes in the gravitational ventilation/perfusion gradient^23^. The improved oxygenation also results from movement of the diaphragm, because, compared with the supine position, the prone position leads to greater excursion in the posterior diaphragm in regions where atelectasis formation and ventilation/perfusion mismatch are most severe during general anaesthesia; furthermore, pulmonary perfusion is more uniformly distributed in the prone position^24^. We assumed that the prone position improves oxygenation during general anaesthesia, regardless of which mechanical ventilatory manoeuvre is used, and further study is required to confirm our results.

Prolonged I:E ratio ventilation and prone positioning improve ventilation/perfusion heterogeneity to produce efficient gas exchange^25,26^. In contrast with some studies, our data showed that PaCO_2_ was increased in the intraoperative period. We permitted the study practitioners to manage the respiratory rate and maintain an end-tidal carbon dioxide tension (EtCO_2_) level between 33 and 36 mmHg. This may have created bias. Additional studies are needed to verify the actual impact of ERV on gas exchange.

The present study had some limitations. Firstly, even though we used a randomisation plan generator, the mean body mass index (BMI) of the ERV group was higher than that of the CRV group. Despite this statistical deficiency, ERV did not confer better arterial oxygenation than CRV in patients in the prone position in the present study. In one study by Kendale and Blitz, an increase in BMI correlated with an increase in intraoperative hypoxaemia risk^6^. Therefore, in the present study, the mean BMI of both groups may have affected arterial oxygenation during the study period. Secondly, our study group did not include morbidly obese patients whose BMI was more than 35.0 kg/m^2^; the purpose of the study was to investigate the effect of prolonged I:E ratio ventilation in overweight and obese patients. Hence, a supplemental study examining prolonged I:E ratio ventilation during spine surgery should be performed in morbidly obese patients.

In conclusion, ERV conferred lower peak inspiratory pressure and higher mean airway pressure than CRV in overweight and obese patients undergoing posterior lumbar spine surgery in the prone position under general anaesthesia. Therefore, ERV may reduce the airway pressure increase in overweight and obese patients undergoing spinal surgery in the prone position. Even though ERV did not confer better arterial oxygenation than CRV in the present study, ERV may be feasible in overweight or obese patients during general anaesthesia. Further study is needed to confirm the impact of ERV on oxygenation in patients with elevated BMI undergoing spine surgery in the prone position.

## Methods

### Subjects

This study was approved by the institutional Ethics Committee (Gachon University Gil Medical Center Institutional Review Board 2016–211, Incheon, Korea) and registered at the US Clinical Trials Registry (NCT02961920). After obtaining written informed consent, we assessed 54 patients scheduled for lumbar spine surgery in our institution between November 2016 and October 2018. Fifty adult patients with an ASA physical status of I or II and a BMI of ≥25 kg/m^2^ were enrolled the study. Patients were excluded if they had severe pulmonary disease (history of chronic obstructive pulmonary disease, asthma, bronchopleural fistula, or pneumothorax), haemodynamic instability, and/or hypovolaemia. The enrolled patients were randomly allocated according to a predetermined sequence to receive an I:E ratio of either 1:2 (CRV; n = 25) or 1:1 (ERV; n = 25). One researcher in our institution who was not involved in the present study generated the allocation sequence without blocking using a randomisation plan generator (http://www.randomisation.com). This study was performed in accordance with relevant guidelines and regulations, including the CONSORT guidelines (Fig. 1).

### Anaesthesia

To induce anaesthesia, intravenous propofol (1.5 mg/kg) and rocuronium (0.8 mg/kg) were administered. Volume-controlled mechanical ventilation was applied, with a tidal volume of 10 mL per ideal body weight (kg), an I:E ratio of 1:2 or 1:1, depending on the group allocation, and no external PEEP. To maintain the EtCO_2_ level between 33 and 36 mmHg during surgery, the respiratory rate was adjusted. To maintain anaesthesia, sevoflurane was used, with an end-tidal concentration of 2–2.5% by volume and a bispectral index score between 40 and 60. Rocuronium (0.2 mg/kg) was injected every hour. The inspired oxygen fraction (FiO_2_) was 0.4, using oxygen and air. Radial artery catheterization was conducted to allow repeated blood sampling and continuous blood pressure monitoring.

### Study design

Standard monitoring during anaesthesia included continuous electrocardiography, systemic arterial pressure, heart rate, EtCO_2_, and peripheral oxygen saturation. We measured intraoperative respiratory mechanics, including peak airway pressure (Ppeak), mean airway pressure (Pmean), plateau airway pressure, and PEEP using the anaesthesia machine (S/5 Avance anaesthetic machine, GE Healthcare, Madison, WI, USA). We also measured arterial blood gas at three time-points: 10 minutes after anaesthesia induction, Tsupine, T30 and T90. We calculated Pdriving, Cdyn, and Cstatic from respiratory data, and D(A-a)O_2_ from arterial gas analysis data. Pdriving was defined as the difference between plateau airway pressure and PEEP. Cdyn and Cstatic were calculated using the following formulas: tidal volume/(Ppeak − PEEP) and tidal volume/Pdriving, respectively. Alveolar oxygen tension was calculated using the following formula: FiO_2_(P_B_ − 47 mmHg) − (PaCO_2_/0.8), where P_B_ is the barometric pressure (760 mmHg).

### Statistical analysis

A previous study reported that, during CRV with an inspired oxygen fraction of 0.4, the mean value (standard deviation) of partial arterial oxygen pressure (PaO_2_) was 181 (28) mmHg in obese patients after placement in the prone position^22^. We assumed a mean PaO_2_ difference of 15% between the CRV and ERV groups. With an α-value of 0.05 and a statistical power of 90%, a sample size of 23 patients was required for each group. Anticipating a 10% drop out rate, we recruited 50 patients.

Continuous variables are expressed as mean ± standard deviation, and categorical variables as number of patients (%). Continuous variables were analysed using the Student’s t-test or Mann–Whitney U test. Categorical variables were analysed using Fisher’s exact test. Serial changes in respiratory variables and haemodynamic changes were analysed using repeated measures ANOVA, followed by Bonferroni correction^27^. After analysing the group × time interaction, we reanalysed serial changes in respiratory variables in each group using repeated measures ANOVA followed by Bonferroni correction. SPSS 21.0 (SPSS Inc., Chicago, IL) was used for statistical analyses.

### Clinical trial number

NCT02961920 (Date of registration: November 11 2016) https://clinicaltrials.gov/ct2/show/NCT02961920?term=NCT02961920&rank=1.

## Data Availability

All data generated or analysed during this study are available from the corresponding author upon reasonable request.
